# Surgical management of the patient living with autism

**DOI:** 10.1016/j.sopen.2019.06.006

**Published:** 2019-07-12

**Authors:** Paige Selvey, Katie Stypulkowski, Steven Waisbren

**Affiliations:** a University of Minnesota Medical School, 420 Delaware St SE, Minneapolis, MN 55455, United States; b University of Colorado Colorado Springs, 1420 Austin Bluffs Pkwy, Colorado Springs, CO 80918, United States; c Minneapolis VA Health Care System, 1 Veterans Drive Ste 2J, Minneapolis, MN 55417, United States

## Abstract

**Background:**

Although 1 in 59 children currently born are diagnosed with autism spectrum disorder (ASD), little is known on how to best manage those patients who require surgical intervention.

The purpose of this study is to (1) investigate the best care strategies for surgical patients living with autism spectrum disorder (2) provide recommendations on how to apply those strategies to clinical practice.

**Methods:**

A literature review was conducted to investigate the best clinical practices for optimizing surgical care for individuals living with autism spectrum disorder. Relevant articles were selected and examined, and individual references from those articles were manually searched using Ovid Medline and Google Scholar.

**Results:**

The wide spectrum of symptoms associated with autism spectrum disorder pose unique challenges for surgical management. Early coordination with the patient and family optimizes the development of an effective care plan. Strategies include identifying triggers for anxiety as well as soothing mechanisms, performing surgery in the morning, completing preoperative paperwork prior to surgery, choosing appropriate analgesia and anxiolytics, and fast resumption of normal routines. Based on these findings a surgical checklist was created to aid in treating the patient with autism spectrum disorder. The checklist provides insight into navigating the surgical experience and emphasizes planning surgical interventions to most effectively fit individual patient needs.

**Conclusion:**

The surgical treatment of those living with autism spectrum disorder poses unique challenges for the health care team. The widespread adoption of such individualized approaches encompassing pre/intra/post-operative will become more important as these children grow into adults with increased needs for surgical services.

## Highlights

• Early coordination optimizes development of an effective care plan.
• Identifying triggers for anxiety as well as soothing mechanisms can be helpful.
• Performing surgery first in the morning decreases deviation from normal routines.
• Completing preoperative paperwork prior to surgery reduces surgical day work.
• Choosing appropriate analgesia and anxiolytics assists in surgical interventions.

## **INTRODUCTION**

Recent incidence rates from the Centers for Disease Control (CDC) estimate that 1 in 59 children currently born are eventually diagnosed with autism spectrum disorder (ASD) [1]. While the challenges of treating those living with ASD are becoming increasingly well described in the medical, psychological, and nursing literature; much less is known on how to effectively manage surgical intervention for those living with ASD. The purpose of this review is to utilize the current knowledge of ASD to present strategies to provide the best possible care for this expanding patient population.

## **METHODS**

A literature review was conducted and papers identified querying Ovid Medline, Google Scholar, and Embase. Search terms included “autism spectrum disorder AND surgery”, “surgical management of autism spectrum disorder”, “anesthesia AND autism”, and “pathophysiology of autism spectrum disorder”. Relevant articles were selected and examined, and individual references from those articles were manually searched using Ovid Medline, and Google Scholar. Articles that directly addressed management of ASD patients in surgery were included. Exclusion criteria included any articles published prior to 1988, articles written in languages other than English, and publications on non-human subjects.

## Autism defined (clinical presentation and diagnosis)

Autism spectrum disorder is a neurodevelopmental syndrome characterized by communication deficits, dependence on routine, and intense focused interests. In 2013 autism was redefined as a single spectrum disorder in the Diagnostic and Statistical Manual of Mental Disorders Fifth Edition (DSM-5) due to the difficulty of differentiating autism, Asperger's Syndrome, and pervasive developmental disorder [2].

The first diagnostic criterion addresses the support needed for social communication. Deficits include both verbal and nonverbal forms ranging from completely nonverbal to a comprehensive vocabulary with awkward or overly literal language to difficulties with pragmatic language. Nonverbal deficits generally include a lack of eye contact, physical gestures, facial expression, or speech intonation. Health care providers may notice that their patients with ASD do not appear to be paying attention because they not making eye contact. However, this may not be the case because one of the first signs of autism is a decline in eye contact. This can be seen as early as 2 months and is, in fact, one of the diagnostic criteria for ASD [3]. People with autism may have difficulty connecting with others that could be due to a lack of interest, a lack of understanding of situational appropriateness, or an inability to reciprocate socially and emotionally [2]. As will be elaborated later, these communication limitations come into play for proper education prior to surgery, pain control postoperatively, and reducing stressful triggers universally.

Surgeons may notice other activities such as repetitive and restrictive behaviors, which can manifest in a multitude of ways depending on the individual's age and developmental level. Some patients might engage in repetitive stereotypic behaviors; such as hand flapping, finger licking, or repetitive speech. People with autism may become fixated on a specific object or be hypersensitive to change; whether that is in the activity they are currently engaged in, a specific process, or a daily routine. Importantly in the surgical setting, these individuals may also display an abnormal sensitivity to specific sensory stimuli. The smell of antiseptics, shining of bright lights, or the beeping of monitors may be particularly disturbing [3].

The DSM-5 includes specifiers to account for some of the accompanying intellectual or language impairments associated with autism including attention-deficit/hyperactivity disorder; developmental coordination disorder; impulse control or conduct disorders; anxiety; depressive or bipolar disorders; tics or Tourette's disorder; self-injury; and feeding, elimination, or sleep disorders [2]. Furthermore, 70% of individuals diagnosed with autism have at least one comorbid psychiatric disorder with 41% having two or more [4].

## Pathophysiology of autism

The cause of ASD remains unknown. The features of autism may be symptoms of some underlying disorder or a disease process on its own. Conceptually, it may be similar to the “diagnosis” of fever, which may have many underlying causes such as an infection, hematoma, transfusion, or abnormal drug reaction. Similarly, no single explanation for ASD has been found. Postmortem studies have helped identify possible pathophysiologic links for autism with almost every section of the brain being linked as a possible cause of ASD. Neurochemistry studies have identified that over 25% of ASD children have elevated levels serotonin, plasma norepinephrine, and cerebrospinal fluid opiate activity [5].

There are no specific biological markers that distinguish ASD. Genetics appears to play some role with 10% of children with autism also having Down syndrome, Fragile X syndrome, tuberous sclerosis, or other genetic or chromosomal disorders. Additionally, parents who have a child with ASD have a 2–18% chance of having a second child who is also affected. Studies on twins do not clarify these issues. Among identical twins, ASD in one twin increases the chance of the other child having the disorder to 36–95%, this chance being 0–31% in non-identical twins [1]. Still, others have linked mitochondrial dysfunction to the development of ASD [6].

Others have attributed low birth weight, advanced parental age, and fetal exposure to valproate as possible associations with ASD [2]. However, the theory that vaccines cause autism has been successfully refuted in multiple well-controlled studies [7], [8]. Even so, it remains a topic of controversy. A very old but still widespread misconception regarding ASD is referred to as the “refrigerator mom” theory. This idea asserts that autism is a result of cold, unloving mothers who display a lack of maternal warmth. Once again, this theory has been widely discarded as understanding of and research into ASD has progressed [2].

## General considerations when treating patients with ASD

The communication and behavioral issues that characterize ASD can create many obstacles in healthcare delivery. ASD patients are often very dependent on consistency to remain emotionally regulated. The possible need to stray from their day-to-day routine and visit an unfamiliar site with different colors, sounds, textures, and odors can cause anxiety and frustration [9]. These new sensory experiences along with the introduction of new caregivers into their lives can all promote anxiety reactions making the surgical experience more taxing for the patient, their families, and healthcare professionals [10].

Social communication impairment poses another difficulty. These patients have difficulty recognizing hand gestures, facial emotions, or tone of voice; which can be important in understanding the procedure and developing a rapport with the provider. Patients with limited verbal abilities may require visual cues such as pictures or objects, or may be able to utilize paper or electronic devices to express themselves [11]. Other studies have shown the importance of having caregivers present whenever possible to help interpret signs of anxiety, pain, and frustration that may not be recognized by their surgical providers [12].

Some have found it helpful to divide patients' behaviors into four categories. (1) The non-compliant patient is prone to emotional outbursts such as temper tantrums, destruction of property, or aggressive actions such as scratching, punching, or head butting. These actions pose particular challenges with wound healing due to reopening of surgical incisions and bleeding from tantrums and/or aggressive activities. (2) The hyperactive patient's behaviors include excessive movements, rummaging in drawers or trashcans, or even running through the halls trying to “elope”. (3) Self-stimulatory actions are twisting of the hands, eye blinking, unusual noises, biting, and repetitive movements such as spinning. (4) Self-injurious behaviors including head banging, pinching or biting oneself, auto-extraction of teeth, and self-induced vomiting may indicate underlying anxiety, depression, or stress [13], [14], [15] . These maladaptive activities may serve as a mechanism to avoid or counteract new stimuli [16].

## Perioperative strategies

Based on the general knowledge of autism, some strategies may be employed to improve the quality of care for the patient and their caregivers during the surgical experience. To date, there have been very few studies examining specific programs in the perioperative care of patients living with ASD. Thus, the peri-operative surgical recommendations in this report are derived from “best practice experience” and knowledge about the general needs of those with ASD.

## Preoperative management

### Non-pharmaceutical strategies

The key to a successful surgical experience, we feel, is preoperative planning with all healthcare providers having a strong understanding of the specific needs and triggers of their particular patient [14], [17]. For younger patients, the surgical team should reach out to others besides the parents. The child's special education teacher, behavioral therapist, or primary physician may be helpful in planning to provide insight into the child's needs. Specifically, the surgical team should ask what stimuli may trigger anxiety or maladaptive behaviors and, conversely, what interventions may be soothing. Healthcare providers should focus on the underlying causes of the various behaviors rather than concentrating on the behaviors themselves.

Other successful strategies include completing paperwork prior to the scheduled OR date, preliminary visits to the hospital/surgical suite to familiarize the patient to the environment, and preoperative telephone surveys to help strategize with caregivers and patients. For children and lower functioning adults, the use of social stories may be helpful in introducing the environment that will be encountered, and the types of personnel that they will be working with. It is important to remember that not all patients with ASD have significant caregivers. As mentioned previously, there is a wide spectrum of ASD presentations with many individuals going unrecognized by casual observers. Further, it is important to recognize that the communication and sensory issues are as real for highly successful people with ASD as is the case with those who are more impaired. Therefore, planning a care strategy and organizing the surgical intervention may involve working with the patient directly. In this case, it is even more valuable to work out a specific plan prior to surgical intervention, as the patient may need more time communicating needs and specific guidelines. Some of this work may be accomplished using a preop telephone survey [10].

Still, a pre-op visit or tour of the facilities along with the necessary paperwork completed in advance has been shown to make the day of the operation run much smoother [11], [14], [18], [19], [20]. For others, however, the prospect of going to a new and unfamiliar place may actually increase anxiety leading up to the surgery. Notably, these same general strategies may also be helpful for a neuro-typical patient.

For instance, ASD patients about to have surgery should be admitted to a separate quiet room with minimal stimuli prior to the planned operation [19]. Closing doors, using “do not disturb” signs, and spot-checking vital signs instead of continuous monitoring may increase compliance and reduce overall anxiety [14]. Additionally, performing the operation as the first case of the day lessens waiting time and starvation periods leading to a better surgical experience for the patient, caregivers, and providers [21].

The same quiet area may also be an ideal location for the premedication of drugs before heading back to the operating room. Once again, caregivers may be the best resource as the administration of medications can be a challenge for these patients and some routines and strategies for medications may have already been worked out at home as somewhat of a ritual [18]. These include what drinks to mix with the medications and how these pills/elixirs are specifically administered.

It may also be helpful to avoid separation from the patients' caregivers as much as possible. In the operating room, the caregiver could be present both during the induction of anesthesia and later when the patient wakes up and recovers [9], [22]. Using the patient's familiar bedding and decorations may also help to reduce anxiety and stress prior to and after surgical intervention [23].

Staff consistency and routine should also be a priority to decrease potential anxiety-provoking situations [20]. Any objects, videos, or pictures from home that the patient is emotionally attached to should be brought to serve as a distraction and to provide comfort during recovery [9], [20].

It is important to emphasize that surgeons and healthcare providers should be educated on the diverse needs of their patients living with autism *before* their operation [24]. They must be armed with strategies for working with uncooperative or combative patients [21].

### Preoperative anxiolysis medication options

In addition to the challenges faced in a normal healthcare visit, those living with ASD may be particularly sensitive to new sensory stimuli such as blood pressure monitoring, rough bedding, beeping monitors, blood draws or tight spaces [25]. All of these experiences may cause significant anxiety that may also be treated with anxiolytic medications. No particular drug has been shown to be superior to the other. However, some recommend administering these medications orally, often disguised in a favorite food or drink.

Midazolam [Versed] (0.5 mg kg^− 1^) [21], is the preferred preoperative anesthetic due to characteristics of rapid onset, short duration, and minimal adverse effects [26]. We usually consider midazolam as an IV medication, however it may be given orally prior to IV insertion [28]. It remains the most commonly used benzodiazepine for premedication, but has a persistent bitter taste even when added to syrup. With oral administration sedative effects are seen within 5–10 minutes and dissipate within 45 minutes. Oral administration is preferable to the nasal route because of burning sensation in the nares. One study found success by mixing oral ketamine and midazolam in the soft drink Dr. Pepper to mask the bitter taste [27].

Diazepam, on the other hand, is offered in flavored syrup or tablets and is absorbed reliably from the gastrointestinal tract. Unfortunately, this drug has a relatively long half-life, which may even be longer in children with immature or dysfunctional livers [29]. In addition, the usual oral dose from 0.1 to 0.3 mg kg^− 1^ has a relatively slow onset of action and must be given 60–90 minutes preoperatively, which may increase the patient's overall stay in the hospital [28], [29].

Ketamine is a useful alternative to benzodiazepines. It is available in a lollipop form, and when administered orally at a dose of 5–8 mg kg^− 1^, sedation occurs within 20–25 minutes leading to calm separation and good induction. This premedication may also be combined with midazolam and administered intramuscularly for the extremely uncooperative patient, which results in effective sedation within 5 minutes. Furthermore, it has a decreased risk for respiratory depression [30]. However, Ketamine has a relatively long duration of action [28]. Side effects include vomiting, agitation, and hallucinations [31].

Clonidine, (2–4 mcg kg^− 1^) an alpha-2 agonist, has been shown to be effective. However, unlike the benzodiazepines; clonidine enhances memory rather than having an amnestic effect. Still, it often initiates sleep within a half hour. Compared to guanfacine it has fast initial burst of activity and a relatively short half-life. One drawback is clonidine's prolonged need for frequent oxygen supplementation, which can provoke anxiety in these patients [28]. Similar to clonidine in mechanism of action, but with fewer side effects, is the relatively new drug, dexmedetomidine [Precedex] [32]. It may be mixed with apple juice at a dose of 5 mcg kg^− 1^.

The choice of which drug to choose needs to be individualized for each particular patient, keeping in mind their unique symptoms of ASD and what medications they may already be taking [28].

### Non-pharmacological management options

Physical restraint may be necessary. Providers typically think physically restraining uncooperative patients should be avoided. Furthermore, older and physically more mature patients may not be easily manipulated and may require the presence of additional trained staff. However, some with autism find deep pressure stimulation to be calming^17^. If restraint does become necessary for the administration of anesthesia, gentle holding by parents and healthcare providers is preferred over devices [25].

## Intraoperative management

After the judicious use of pre-operative anxiolytics, operative strategies should be employed to make the postoperative care as smooth as possible. Tactile aversions can make the administration of anesthesia with injections or even with an oxygen mask problematic [19], [21]. Thus, the choice of general vs. regional/local anesthesia may be affected by the patient's behaviors and their ability to cooperate [33]. Surgeons may also consider limiting the use of Foley catheters, NG tubes, and surgical drains; which all may be particularly traumatizing for those with ASD. Skin closure techniques using Dermabond or Steri-strips may be preferable because their use obviates the need for suture/staple removal.

## Postoperative management

A prolonged stay should be avoided because blinking lights, new odors, and beeping monitors in the PACU are often stressful and may lead to challenging behaviors [9], [22]. Aspects of surgical care that are usually routine to the surgeon, such as dressings and bandages covering the surgical area, may also be seen as intolerable to the patient with autism [25]. Tasks such as changing dressings, taking vital signs, or bathing should be completed by the caregiver or someone familiar to the patient whenever possible.

Management of postoperative pain is another difficult task in this patient population and has been an area of confusion in the past [9]. A common misconception is that patients with autism have reduced pain sensitivity because they do not demonstrate or communicate discomfort in the same way as their neuro-typical peers. In one-study, Arnold et al., found that those in the ASD group were two times less likely to report pain than their peers [33]. However, recent investigations using other markers of pain such as heart rate, facial expression, and levels of beta-endorphins suggest that patients with autism are just as reactive to painful stimuli as children without the disorder; but do not *express* their discomfort in the same way [13], [34].

Pain scales and behavioral observation-based assessment may be useful [20]. The Child Facial Coding System utilizes various facial expressions such as lowered brows, squinted eyes, and open lips to recognize pain [35], [36]. A challenge with these tools is that they have not been validated in children with ASD. Another possible tool is the Noncommunicating Children's Pain Checklist [37]. This relatively lengthy process examines vocal, social, facial, and daily activities to assess a patient's pain control through an accessible 30-question survey during a two-hour timespan [38]. Once again, these instruments may not be as valuable with those with ASD because of the heterogeneity of these responses with those living with ASD. The Observational Scale of Behavioral Distress is popular in the pediatric population. It examines 8 defined behaviors including crying, screaming, and verbal/physical resistance, which have been associated with distress, anxiety, and pain [39], [40], [41].

Lastly, probably no one knows the patient better than their parent or primary caregiver [20], [34], [42]. Parents are best at recognizing subtle signs of discomfort including grimacing, moaning, shaking, and hunched walking. Contact should be maintained between the healthcare team and the caregivers until the recovery process is complete. Other post-op strategies include using physical therapy and occupational therapy based out of the home, rather than at an outside institution to minimize routine disruption.

## ASD surgical check list

Based on these data we developed a surgical checklist that may be helpful in navigating the surgical experience. We wish to emphasize that patients are unique and, thus, surgical planning should be individualized to most effectively fit their needs. It is notable that many of these strategies may also be helpful for the neurotypical patient.

**Figure:**
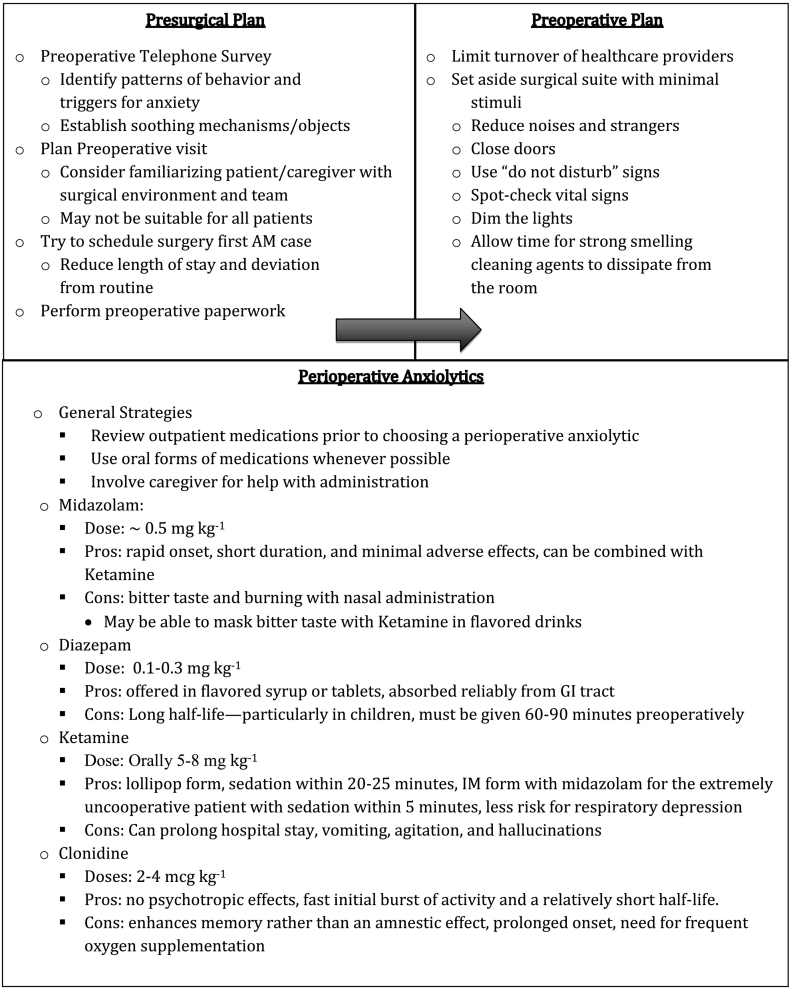

**Figure:**
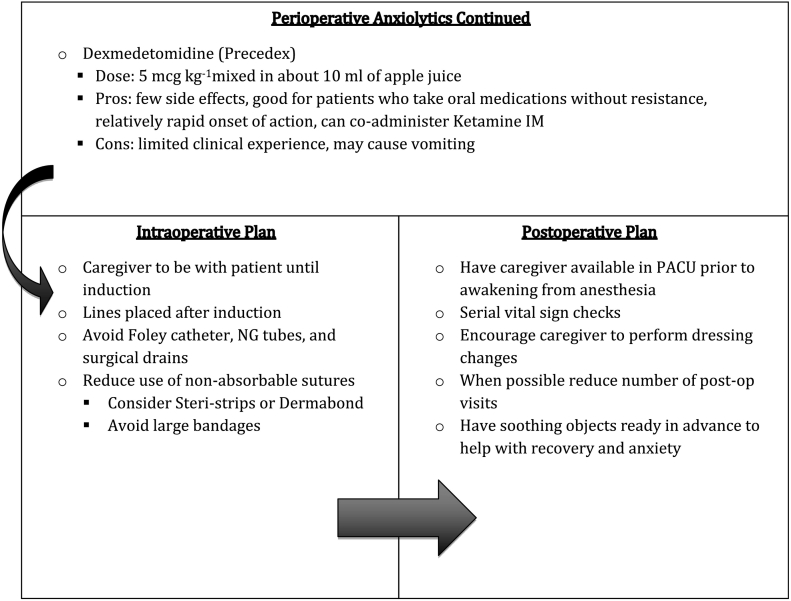

**Figure:**
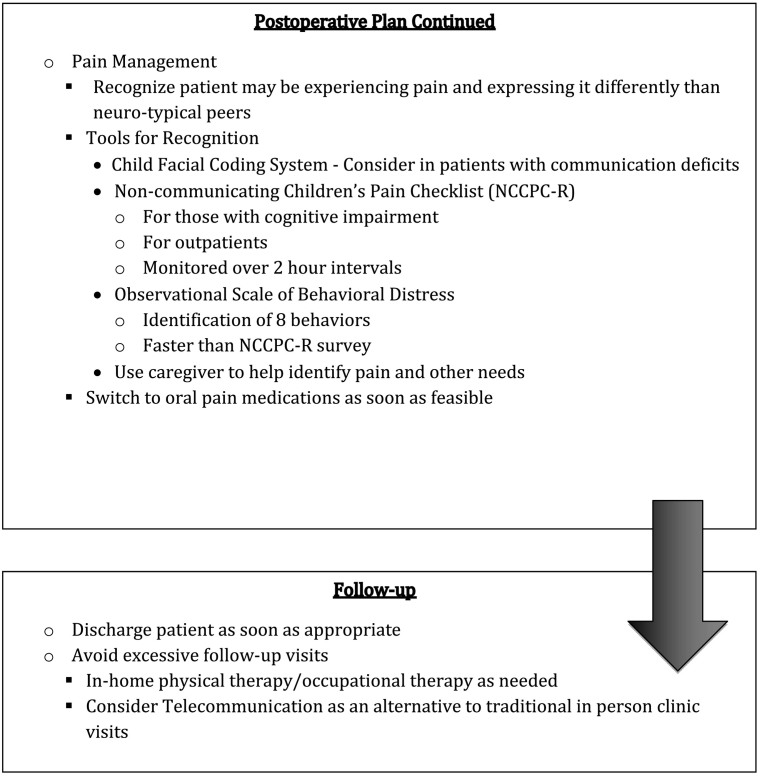

## Further research—proposed areas for further investigation

These recommendations on surgery for those living with ASD are based on a relative paucity of prospective data. The efficacy of this checklist could be fairly easily examined comparing the quality of care delivered both before and after the checklist/interventions are employed. Measured endpoints could include (1) Complication rates (2) Length of stay (3) Time to resumption of usual activities (4) Patient/caregiver satisfaction using a survey with a 1–10 scale asking (a) How distressing was the surgical experience for the patient? (b) How distressing was the surgical experience for the caregivers? (c) Overall, how would this surgical experience be rated? In addition, an open-ended question that may provide healthcare providers with additional insight should be included in this survey that asks, “What advice do you have for the surgical team to improve the care for those living with autism?” Lastly, other studies could be initiated to help determine which preoperative anxiolytics work best for those living with ASD.

In conclusion, despite the very high prevalence of ASD in our society, there remains significant deficiency of previous research on the surgical management of these patients. The wide spectrum of symptoms associated with ASD pose unique challenges for the surgical management of these patients and may complicate even “routine” procedures. Early contact with the patient, family, and/or caregivers is the best way to facilitate an understanding of the patient's needs and effectively develop a plan of care. Strategies unique to the individual with ASD should be followed closely throughout the **entire** surgical process to return them to their normal routine as soon as possible. Familiarizing healthcare providers on the treatment, appropriate interventions, and services for those living with autism will become even more important as the extremely large number of children diagnosed with autism grow up into adults with increased needs for surgical services.

## Disclosures

**Author Contribution**

Paige Selvey B.S. researched and helped write the manuscript.

Katie Stypulkowski M.A researched for the paper.

Steven Waisbren M.D., Ph.D. directed and participated in the research and writing of the manuscript.

## Conflict of interest

This research did not receive any specific grant from funding agencies in the public, commercial, or not-for profit sectors.

## Funding sources

No funding agencies in public, commercial, or not-for-profit sectors.
